# Somatic inhibition by microscopic magnetic stimulation

**DOI:** 10.1038/s41598-021-93114-x

**Published:** 2021-06-30

**Authors:** Hui Ye, Lauryn Barrett

**Affiliations:** Department of Biology, Quinlan Life Sciences Education and Research Center, Loyola University Chicago, 1032 W. Sheridan Rd., Chicago, IL 60660 USA

**Keywords:** Biophysical models, Neurophysiology, Biomedical engineering, Electronics, photonics and device physics, Extracellular recording, Intracellular recording, Transcranial magnetic stimulation

## Abstract

Electric currents can produce quick, reversible control of neural activity. Externally applied electric currents have been used in inhibiting certain ganglion cells in clinical practices. Via electromagnetic induction, a miniature-sized magnetic coil could provide focal stimulation to the ganglion neurons. Here we report that high-frequency stimulation with the miniature coil could reversibly block ganglion cell activity in marine mollusk *Aplysia californica*, regardless the firing frequency of the neurons, or concentration of potassium ions around the ganglion neurons. Presence of the ganglion sheath has minimal impact on the inhibitory effects of the coil. The inhibitory effect was local to the soma, and was sufficient in blocking the neuron’s functional output. Biophysical modeling confirmed that the miniature coil induced a sufficient electric field in the vicinity of the targeted soma. Using a multi-compartment model of *Aplysia* ganglion neuron, we found that the high-frequency magnetic stimuli altered the ion channel dynamics that were essential for the sustained firing of action potentials in the soma. Results from this study produces several critical insights to further developing the miniature coil technology for neural control by targeting ganglion cells. The miniature coil provides an interesting neural modulation strategy in clinical applications and laboratory research.

## Introduction

Abnormal neuronal activity underlies numerous neurological diseases. Pharmacological interventions have been developed to inhibit these activities. Unfortunately, drugs are usually slow in action and sometimes causing unwanted side effects such as systemic toxicity^1^. Electric stimulation to the neural tissue provides a quick and reversible method in control neural activity. Among many methods for neural modulation with electric currents, high-frequency stimulation (HFS, frequency between several hundred and several kilohertz) has been explored as a reliable method for neural inhibition. For example, high-frequency electric stimulation can provide blockage to axonal conduction in peripheral nerves^2,3^. Axonal and synaptic failure were also observed during deep brain stimulation of subthalamic nucleus (STN) using high-frequency pulses, which suppressed the firing rate, oscillations, synchrony, and information transfer from STN to its synaptic targets^4^. Blocking abnormal neural activity with high-frequency electric field is widely used for clinical conditions, such as pain^5,6^ and detrusor-sphincter dyssynergia^7^.

Neuron clusters, or ganglia neurons, are found throughout the peripheral nervous systems in humans and invertebrates. Electric stimulation to the ganglion cells provides a rapid and reversibly control of ganglion cell activity. For example, neuropathic pain is associated with physiological changes and hyperexcitability of dorsal root ganglion (DRG) neurons^8^. High frequency electric stimulation on the ganglion cells provided analgesic effects^9^. There are several advantages assocaited with ganglion cell stimulation. First, focal activation/deactivation of the clustered neurons by the electric stimulation could avoid passing axons or neurons located far away, therefore improve the specificity and outcome of stimuli. Second, because there is a high sodium channel density in the axon initial segment, the threshold of spike initiation and inhibition is usually around the proximal axon under electric stimulation^10^. This leads to less power being necessary for stimulation. As a matter of fact, DRG stimulation requires much less power than spinal cord stimulation for pain control^11^. Third, since stimulation is at the point where action potentials are generated, it is less likely to induce antodromic action potentials in the fiber.

Electric current can be delivered via an electrode, or through magnetic coils using electromagnetic induction^12,13^. In comprising with the electrode-based technology for electric current delivery, stimulation via a magnetic coil has several advantages. First, magnetic coil stimulation could be non-invasive, since the coil does not need to be in direct contact with the target tissue^13^. Second, since biological matters have negligible magnetic susceptibility, they allow magnetic fields to penetrate through the tissue undiminished. Therefore, the electric current generated by the coil could be more predictable than those delivered by an electrode in the tissue. Previously, Parson et al. stimulated the individual ganglion neurons in freely behaving animals (*Aplysia)* using attached fine wire on the ganglion, and found unpredictable, high current induced noxious responses in the animals^14^. Third, if necessary, the magnetic coil could also be implanted under the cover of soft material^15^, which minimize the foreign body reaction associated with implantation.

Despite the advantages of using magnetic fields for ganglion neuron stimulation, there have been no attempts using a magnetic coil to inhibit ganglion cell activity. This is mainly due to several technical challenges. First, fabrication of coils that can deliver strong electric field, specifically to the small-sized ganglion is challenging. Second, it is not clear what kind of magnetic field can cause ganglion inhibition. Third, if high-frequency stimuli were to be used, it is difficult for the large-sized coil to generate time-varying magnetic field at high frequency. Finally, potential noise and thermal effects caused by the coil could be a challenge in interpreting the outcome of magnetic stimulation.

Recent developments of the micro coil technology provide opportunities to explore the inhibitory effects of the magnetic stimulation to ganglion neurons. These coils can be manufactured to match the size of the targeted ganglion for focal stimulation. The coil can also be implanted under the cover of biocompatible, soft material, to avoid inflammatory and immune responses due to the direct contact with the tissue^16,17^. Evidence has shown that the miniature coil could be used to control retinal ganglion neuron activity using high amplitude pulses^18^. Recently, we reported that a miniature size magnetic coil, by generating high-frequency electric field, blocked axonal conductance in unmyelinated axons^19^. Similar stimulation parameters can also be used to block hyperactive circuity activity in the hippocampus in mice^20^.

The marine mollusk, *Aplysia californica*, provides an appealing system for the study of ganglion cell inhibition by the miniature coil technology. The buccal ganglion in *Aplysia* contains large interneuron and motor neurons that control the feeding behavior of the animal. Previous studies have shown that single buccal ganglion neuron activity could be selectively controlled with electric currents in vitro^21^ and in vivo^22^, with an electrode positioned close to the ganglion. Since these buccal neurons have identified soma location, axonal projection, and dedicated neuronal functions^21–27^, modulation of their activities can potentially be used to control animal behavior^28,29^. Furthermore, a detailed multi-component NEURON model of the buccal neuron has been built for the study of the neuronal response to electric stimulation^21^ and magnetic coil stimulation^19^.

To explore whether the high frequency, miniature coil stimulation could indeed block ganglion cell activity and eliminate its functional output, we used a combined approach of electrophysiological experiments and computational simulation. We delivered strong, high frequency currents into a miniature coil, whose size matched the size of the targeted buccal ganglion. The miniature coil stimulation led to a quick and reversible blockage of the ganglion neurons, regardless of the firing frequency of the neuron, the presence of the ganglion sheath, or the changes in the neuron’s micro-environment. To understand the mechanisms of ganglion cell inhibition by the miniature coil, we estimated the magnetically-induced electric field in the vicinity of the targeted neuron and applied this induced electric field to a multi-compartment model of *Aplysia* neuron using NEURON simulation. To investigate the underlying mechanisms of coil-induced ganglion cell inhibition, we analyzed the detailed ion channel dynamics for the sustaining of the somatic action potentials. High frequency magnetic stimulation caused the sodium channel inactivation, leading to the failure of sustaining the somatic action potentials.

## Results

**Figure 1:**
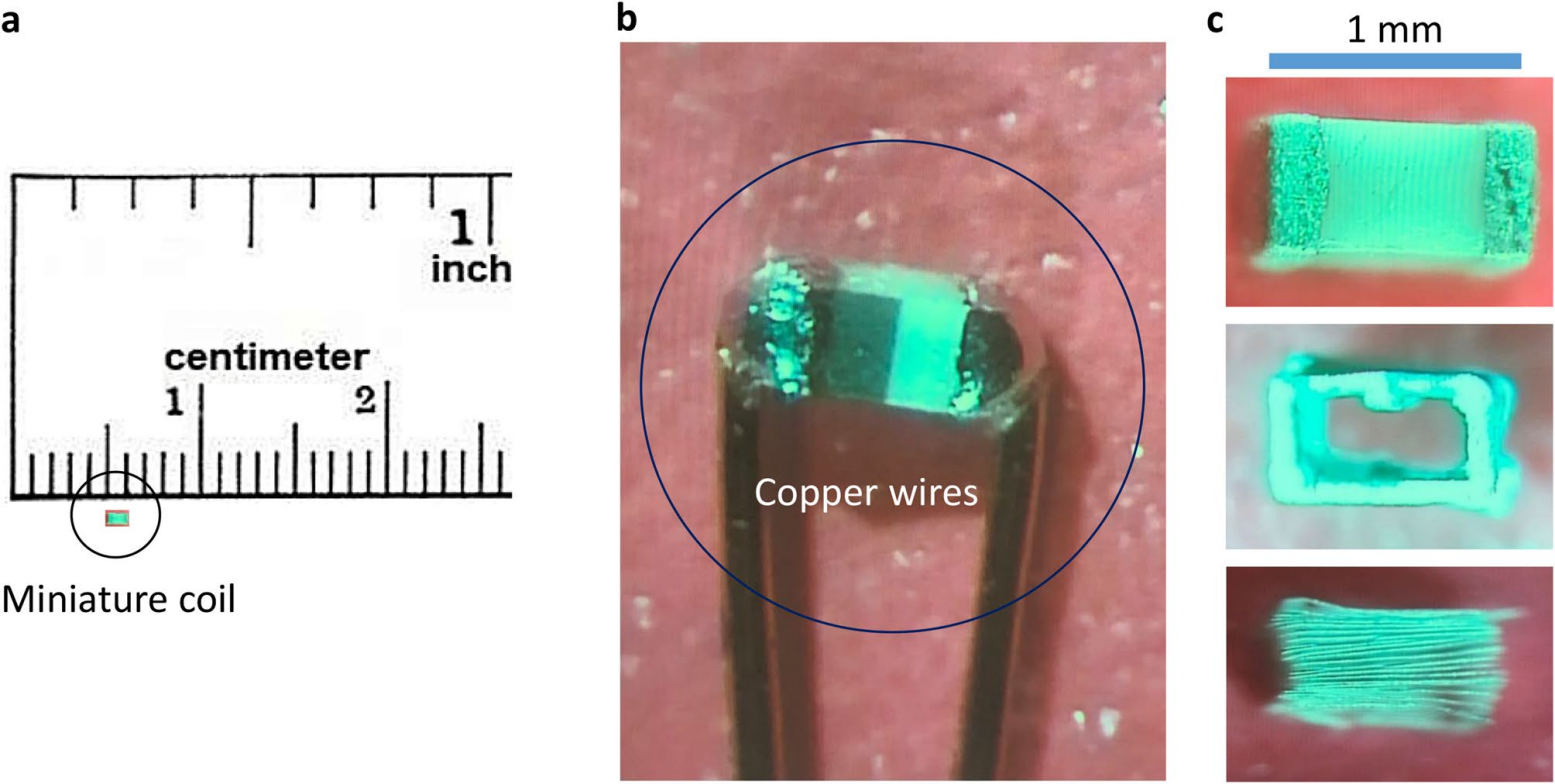
The miniature coil used for the electrophysiology experiments. (**a**) The size of the coil was compared to a ruler. (**b**) The two leads of the coil were soldered to two copper wires for electric current delivery. (**c**) The internal structure of the coil was revealed by chemical removal of the ceramic cover. The coil contained 20 loops of fine wires in rectangular shape.

In this paper, we describe the usage of a sub-millimeter magnetic coil (Fig. 1) for buccal ganglion cell inhibition in *Aplysia californica*, by positioning the coil near the cell bodies of the ganglia. Miniature coil provided focal stimulation to the ganglion cells, thanks to its small size that can match the dimension of the ganglion. We seek to (1) understand if the coil, by generating fast pulses, can inhibit neurons that are firing at various frequencies; (2) Test if neuronal firing under conditions of abnormal ion concentration can also be inhibited by the miniature coil; (3) Experimentally demonstrate that soma inhibition by the coil indeed prevents the functional output of the motor neurons in the buccal ganglion. (4) Demonstrate that the inhibitory effects of the coil can be applied across through the ganglion sheath. (5) Explore the ion channel mechanisms underlying soma inhibition using a multi-compartment NEURON model.

### High-frequency stimulation delivered by the miniature coil suppressed buccal neuron activity, regardless of the neuron’s firing frequency

**Figure 2:**
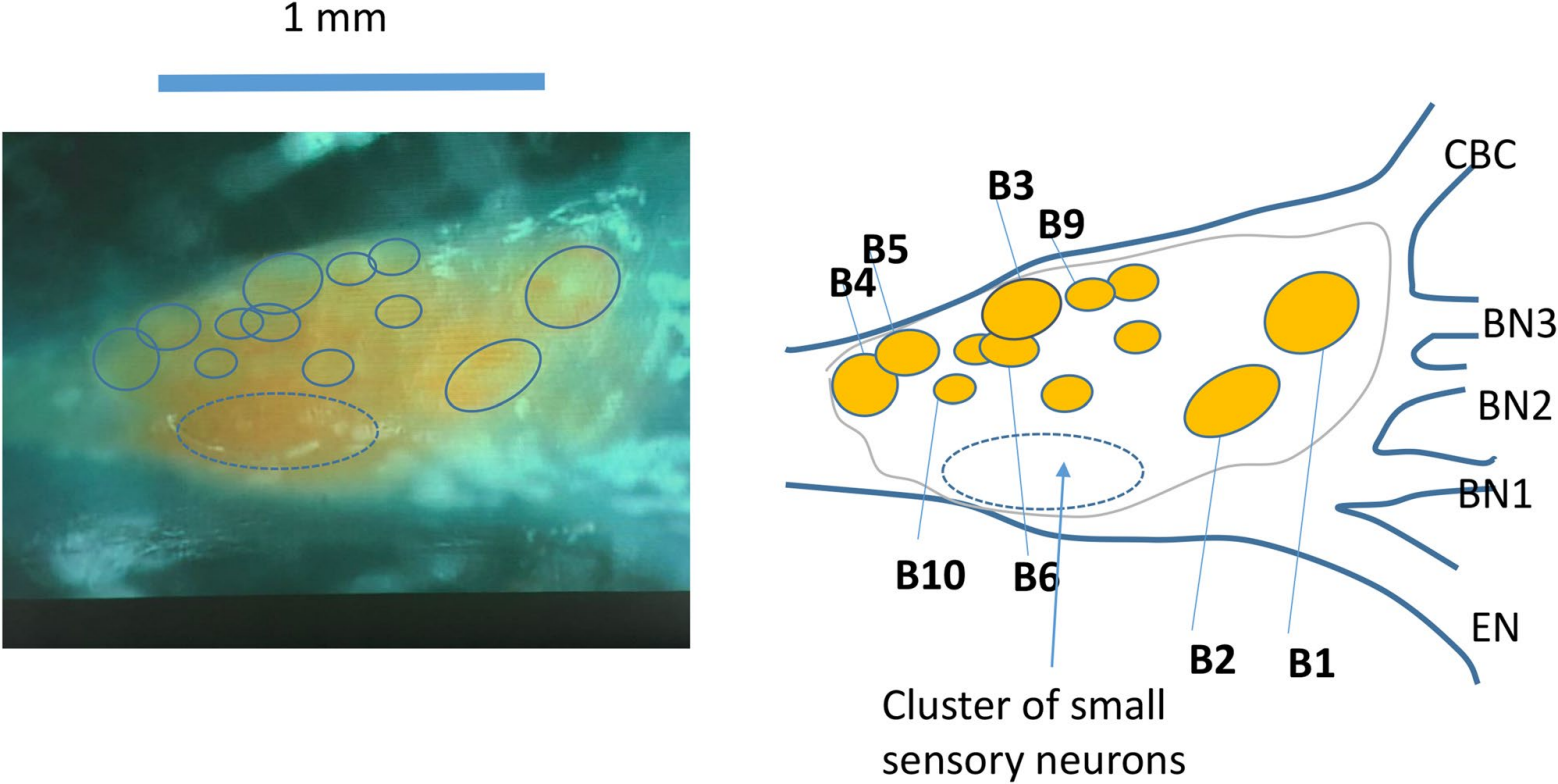
Anatomy of the buccal ganglion. Left picture shows one buccal ganglion. The right schematic was drawn based on the left picture. The picture and schematic together indicated the locations of several identified interneurons (B4 and B5), motor neurons (B3, B6, B9, B10) whose axons innervate buccal nerve 2 (BN2), and a cluster of small sensory neurons. EN: esophageal nerve. BN1: buccal nerve I; BN2: buccal nerve II. BN3: buccal nerve III. CBC: cerebro-buccal connection.

**Figure 3:**
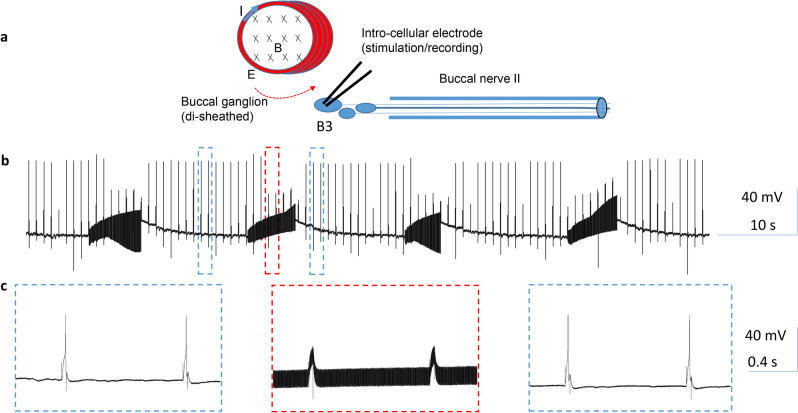
High-frequency magnetic stimulation inhibited buccal ganglion neurons firing at a low rate. (**a**) Experimental setup for the magnetic stimulation of the buccal neurons. The buccal ganglion was de-sheathed. An intracellular electrode was inserted into the B3 motor neuron for stimulation and recording. A miniature coil was positioned on top of the buccal ganglion for magnetic stimulation. I: electric current flow inside the coil. X: direction of the magnetic field generated by the coil current. Red arrow indicates the induced electric field by the coil. (**b**) Single action potentials were generated at 1 Hz by 4 ms current pulses delivered to the B3 soma. Coil stimulation reversibly suppressed these action potentials. (**c**) Expanded traces in (**b**).

In this set of experiments, the buccal ganglion (Fig. 2) was di-sheathed, and the buccal neuron B3 was intracellularly stimulated and recorded (Fig. 3a). A short pulse of current was injected into the B3 soma to elicit a single action potential at a slow rate of 1 Hz. The miniature coil was positioned next to the recorded soma. 400 Hz, biphasic square pulses were delivered into the coil via a power amplifier. The coil stimulation rapidly and reversibly blocked the 1 Hz firing of the B3 neuron (Fig. 3b, n = 5). The stimulation-induced noise could also be recorded by the intracellular electrode. Similar inhibitory effects of the coil on neuronal activity were also observed from other buccal ganglion neurons, such as B4/B5 interneurons (n = 3), and B6 neurons (n = 3). These results suggest that the miniature coil provided a generic, inhibitory effect on the population of neurons in the buccal ganglion.

**Figure 4:**
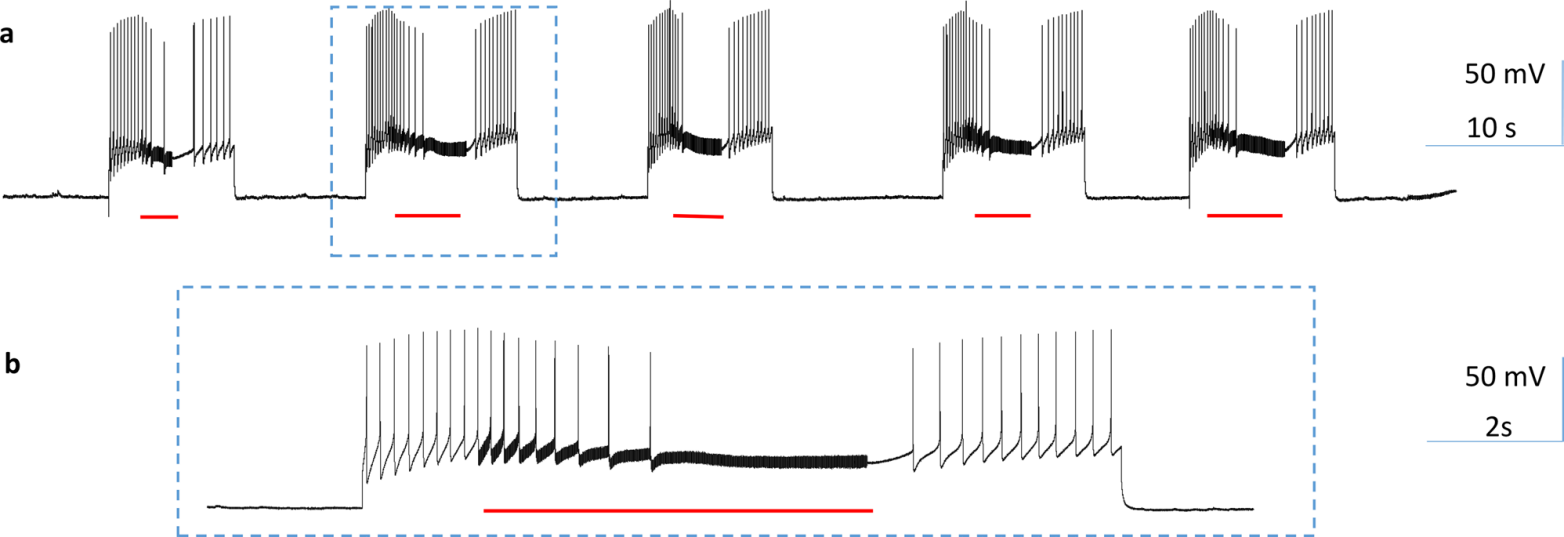
High-frequency magnetic stimulation inhibited buccal ganglion neurons firing at a high rate. Position of the miniature coil was as in Fig. 3a. (**a**) A depolarization current was injected into the soma of the B3 neuron to elicit a train of high frequency (5–7 Hz) action potentials for approximately 10 s. Magnetic stimulation reversibly blocked these action potentials. (**b**) Expanded traces in (**a**).

To evaluate the capability of the coil in blocking the neuron firing at a relatively higher frequency, we depolarized the B3 neuron with a prolonged (about 10 s) DC current, which elicited action potentials at a frequency up to 5–7 Hz. Then the coil was turned on and the 400 Hz, bi-phasic pulses were applied to the coil for approximately 5 s. Coil stimulation caused an initial decrease in the size and frequency of the action potentials, followed by a complete blockage of the action potentials. When the coil stimulus was withdrawn, the neuron resumed its firing of action potentials (Fig. 4, n = 5). In conclusion, high-frequency stimulation at 400 Hz by the miniature coil could reversibly block the neuron firing, regardless of the firing frequency of the neuron.

### Neurons firing under in vitro pathological condition could be inhibited by the miniature coil

**Figure 5:**
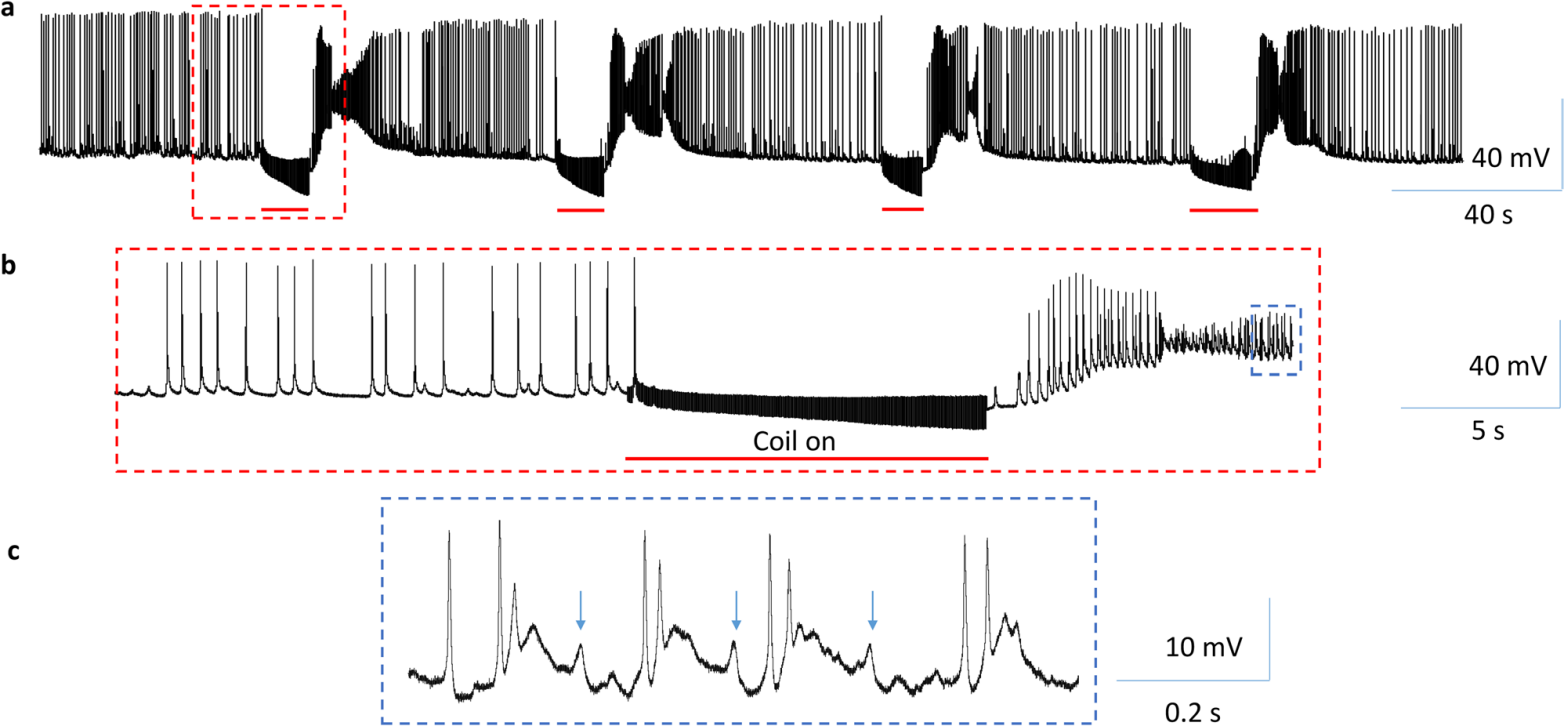
High-frequency magnetic stimulation inhibited somatic neuron activity in a high K^+^ solution. Positon of the miniature coil was as in Fig. 3a. (**a**) Hyperactivity in the B3 neuron was elicited by high K^+^ *Aplysia* saline. 400 Hz stimulation by the miniature coil rapidly and reversibly eliminated the neuron activity. (**b**) Expended trace in (**a**). (**c**) Expanded trace in (**b**), showing increased excitatory events (arrows) after coil stimulation.

The micro-environment of single neurons changes frequently in physiological and pathological conditions. For example, fluctuation of extracellular potassium was observed in the brain tissue in seizure^30^. An increase in K^+^ concentration was observed in hemiparkinsonian rats during deep brain stimulation^31^. Increased K^+^ concentration could lead to individual neuron excitation and network excitability^32^. In normal physiological conditions, *Aplysia* neurons were immersed in the *Aplysia* saline with a K^+^ concentration of 10 mM. To investigate if neurons in the perturbed microenvironment could still respond to magnetic inhibition, we increased the K^+^ concentration to 14.7 mM, by applying additional high K^+^ *Aplysia* saline to the buccal neurons. It took roughly 10 min before the recorded neuron to fire spontaneous action potentials (at around 3 Hz) after the solution been changed. When a steady rate of firing was observed, we applied 400 Hz high-frequency magnetic stimulation to the B3 neuron (n = 4), which instantly blocked the firing of the neurons (Fig. 5). When the coil stimulation was terminated, the neuron recovered and demonstrated a high-frequency firing profile. We also observed increased synaptic events and a plateau of depolarized membrane potential post-stimulation (Fig. 5b,c). This is likely due to the rebounded excitatory input from other buccal neurons that synaptically connected to the B3 neuron, after an overall inhibition of the ganglion activity by the magnetic stimulation.

### Magnetic stimulation eliminated the output from the coil-targeted neuron

According to the principles of current conservation, an electric current that is used to inhibit one segment of the neuron could provide excitation effects to the far sites of the stimulus^33^. It has been observed that high-frequency electric current that suppressed somatic activity could generate excitation of the axon^34^. If this would happen in our experiments, it will compromise the overall inhibitory effects of the miniature coil on the neuron.

Large cell bodies of identified neurons B3, B6, B9, and B10 in the buccal ganglion send axons to the buccal nerve II (BN2)^35^, which innervate the I1/I3 jaw muscle to generate feeding behavior in *Aplysia*^28,29^. The firing of these neurons generates large spikes in the extracellular recordings from the BN2^23,28^. Simultaneously monitoring the soma activity and its axon activity, therefore, provide reliable monitoring of the functional output of these neurons under magnetic inhibition.

**Figure 6:**
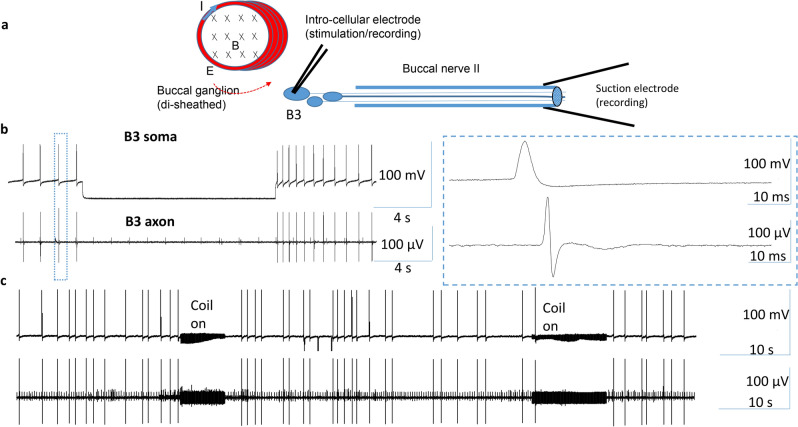
High-frequency magnetic stimulation on the soma inhibited functional output of the B3 motor neuron. (**a**) Experimental setup for the magnetic stimulation of the B3 neuron and assessment of its functional output. (**b**) Spontaneous B3 activity recorded in the soma and the axon demonstrated a one-to-one relationship. The soma action potential led the axonal action potential by approximately 6 ms. Hyperpolarization of the soma eliminated action potentials in both the soma and the axon. (**c**) Magnetic stimulation blocked spontaneous action potentials in the soma, leading to a suppressed activity in the distal axon.

To test the hypothesis that the focal somatic inhibition by the magnetic coil could prevent the motor output from the motor neuron, we applied an intracellular electrode to the B3 soma, and an extracellular suction electrode to the distal end of the BN2, while the B3 soma was stimulated by the miniature coil (Fig. 6a). Dual recording from the soma and the BN2 demonstrated a one-to-one relation, with soma activity leading the axon activity by a few milliseconds. Hyperpolarization of the B3 soma eliminated both somatic and axonal action potentials (Fig. 6b). High-frequency magnetic stimulation of the B3 soma eliminated action potentials in the soma and in the distal axon (Fig. 6c, n = 4). In conclusion, the output of the B3 motor neuron was blocked by the coil-induced inhibition specifically to the B3 soma.

### Miniature coil inhibited the buccal neurons through the ganglion sheath

**Figure 7:**
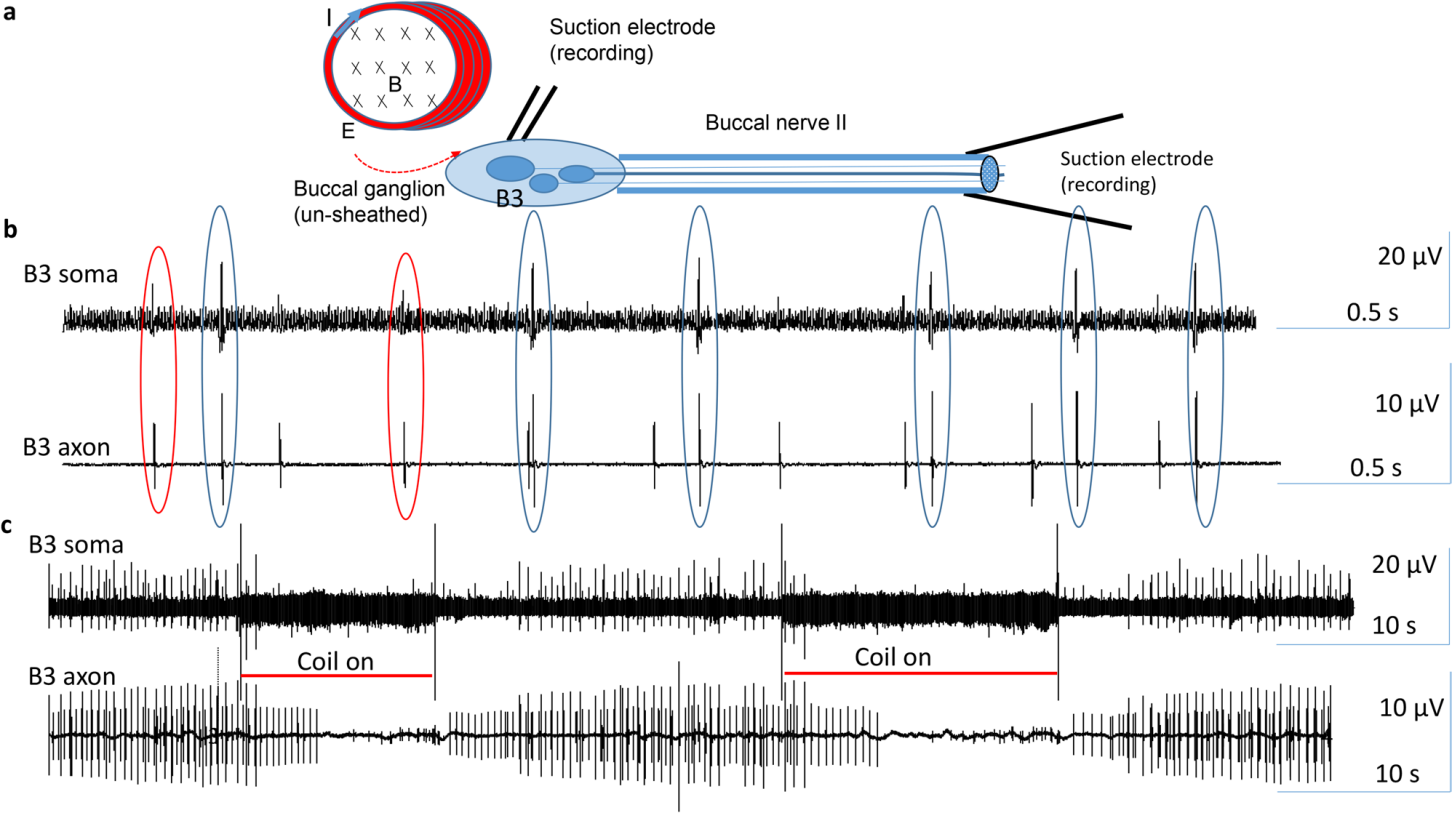
Trans-sheath inhibition of the soma activity by the magnetic stimulation. (**a**) Experimental setup and orientation of the coil to the buccal ganglion. An extracellular electrode was positioned ganglion sheath, right above the soma of the B3 neuron in the un-disheathed buccal ganglion. Another suction electrode was applied to the distal end of the BN2. (**b**) One-to-one relationship between the soma and axon activities were observed in two neurons (blue and red circles). (**c**) Magnetic stimulation on the buccal ganglion eliminated the action potentials in both the soma and axon in both neurons.

**Figure 8:**
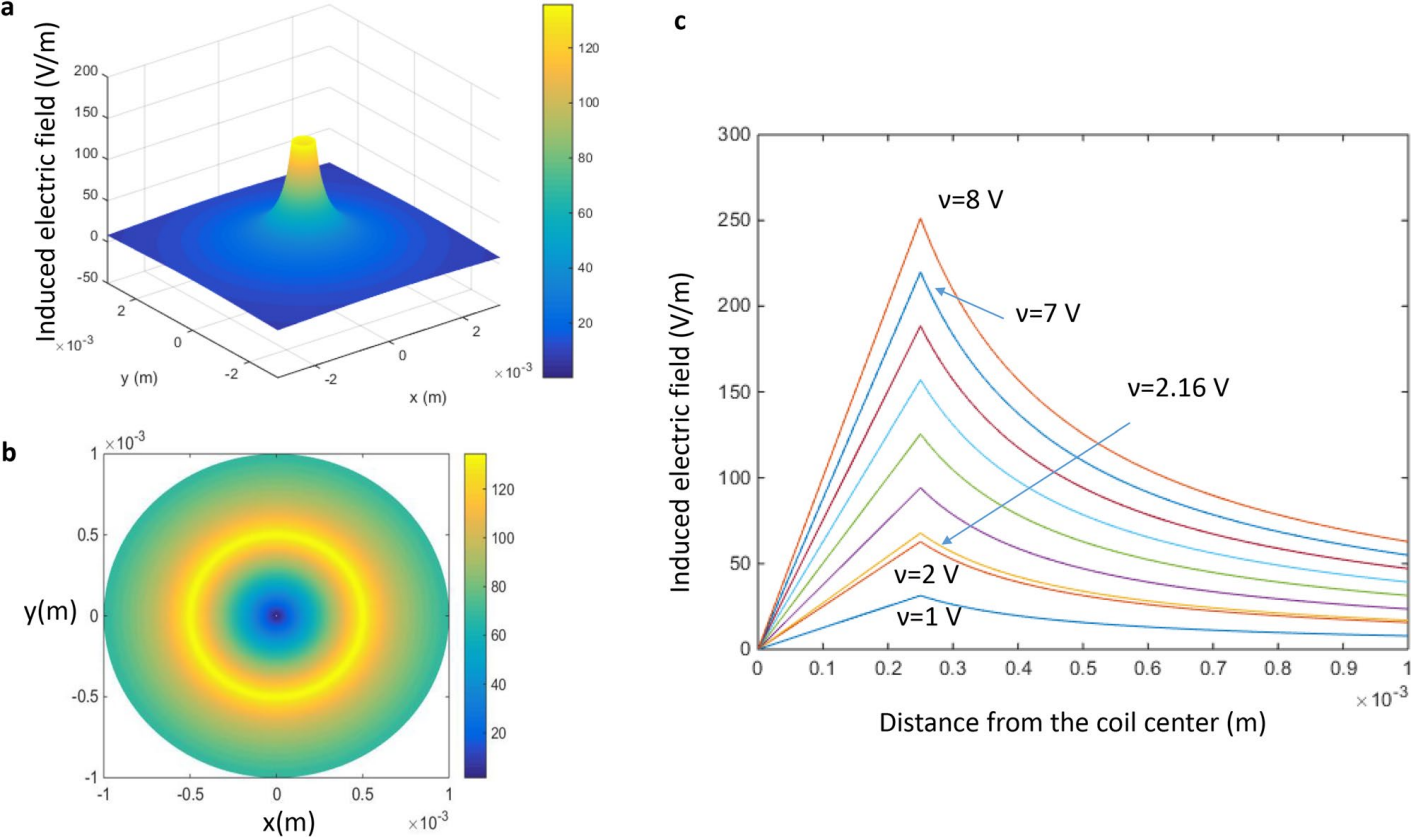
The intensity of the electric field induced by the magnetic coil. (**a**) Distribution of the induced electric field around the coil in a 3D plot. (**b**) Distribution of the induced electric field around the coil in a 2D plot. (**c**) Induced electric field intensity as a function of the distance to the coil center at various voltage across the two ends of the coil. Different colors represented increased voltages in the coil (1–8 V). At 1 mm, the intensity of the E is approximately 20 V/m when the measured coil voltage is 2.16 V, the measured coil voltage for the electrophysiology experiments.

One advantage of inducing an electric field in the buccal ganglion via electromagnetic inductance is that the induced electromagnetic field can penetrate the dielectric membrane without attenuation^13^, therefore controlling a neuron non-invasively. We, therefore, hypothesize that the coil will be equally effective in inhibiting neurons under the ganglion sheath. Unfortunately, it is impossible to record from neurons covered by the ganglion sheath using the intracellular technique. Previous studies have developed an extracellular technology to record soma activity in vitro^21,23^ and in vivo from behaving animals^22^, in which a small pipette electrode has been positioned on the ganglion sheath, above the targeted neuron. Since extracellular activity recorded with this method are relatively small and noise-sensitive, it may be contaminated by the coil-generated noise. We, therefore, apply another suction electrode to the distal axon of the neuron (Fig. 7a), which was approximately 2 cm away from the coil and the ganglion. Since induced electric fields decay quickly (Fig. 8), this suction electrode recorded minimal noise during coil stimulation. More importantly, it monitored the output of the motor neuron B3 to the jaw closure muscle I1/I3 in *Aplysia*^28,29^.

We applied dual extracellular recording from both the B3 soma and the BN2 (Fig. 7b), which contained the axons of B3^23,25^. There is a one-to-one relationship between the largest spikes in the BN2 recording and the B3 activity (blue circles in Fig. 7b). High-frequency coil stimulation suppressed B3 activity in both the soma and in the axon (Fig. 7c). The soma electrode also recorded the 2^nd^ largest spikes (red circles in Fig. 7b), which might be due to the activity of the neurons next to B3, such as B6 and B9^23^ (Fig. 2). Coil stimulation also suppressed these neurons under the cover of the ganglion sheath. Similar results were obtained in four different preparations. In conclusion, trans-sheath stimulation by the miniature coil was equally effective in soma inhibition.

### Computation of miniature-coil induced electric field around the soma

We used a biophysics model to estimate the intensities of the induced electric fields generated by the coil in the vicinity of the buccal neurons. For simplicity, we modeled the miniature coil as an infinitely long, circular cylinder with a radius of 250 µm. Taking into account the coated layer of the miniature coil (approximately 50 µm in thickness), we estimated that the center of the coil was about 1 mm away from the soma.

Time-varying current inside the coil induced an electric field around the coil, which was calculated by Faraday’s law. The induced electric field was the greatest close to the coil. Outside the coil, the induced electric field decayed quickly as a function of 1/r, where r was the distance between the coil center and its target (Eq. 2, Fig. 8). For the 10 V pulses used in the experiments, they generated a 2.16 V voltage across the two coil leads. Using Eq. (2), we estimated that the intensity of the induced electric field was approximately 20 V/m in the recorded neuron.

Previous studies suggest that the intensity of the electric field played a significant role in controlling neuronal activation^36^. An electric field of 5–10 V/m was sufficient in the suppression of epileptic-form activity in rat hippocampal slices^36^. Electric fields at 10–20 V/m have been shown to modulate neurone-firing patterns of Purkinje and stellate cells in the turtle cerebellum^37^ or in the guinea-pig hippocampus^38^. Therefore, the intensity of the electric field in our experiments was within the reported range for modulating neural activity.

### NEURON model confirmed somatic inhibition by the high-frequency magnetic stimulation

**Figure 9:**
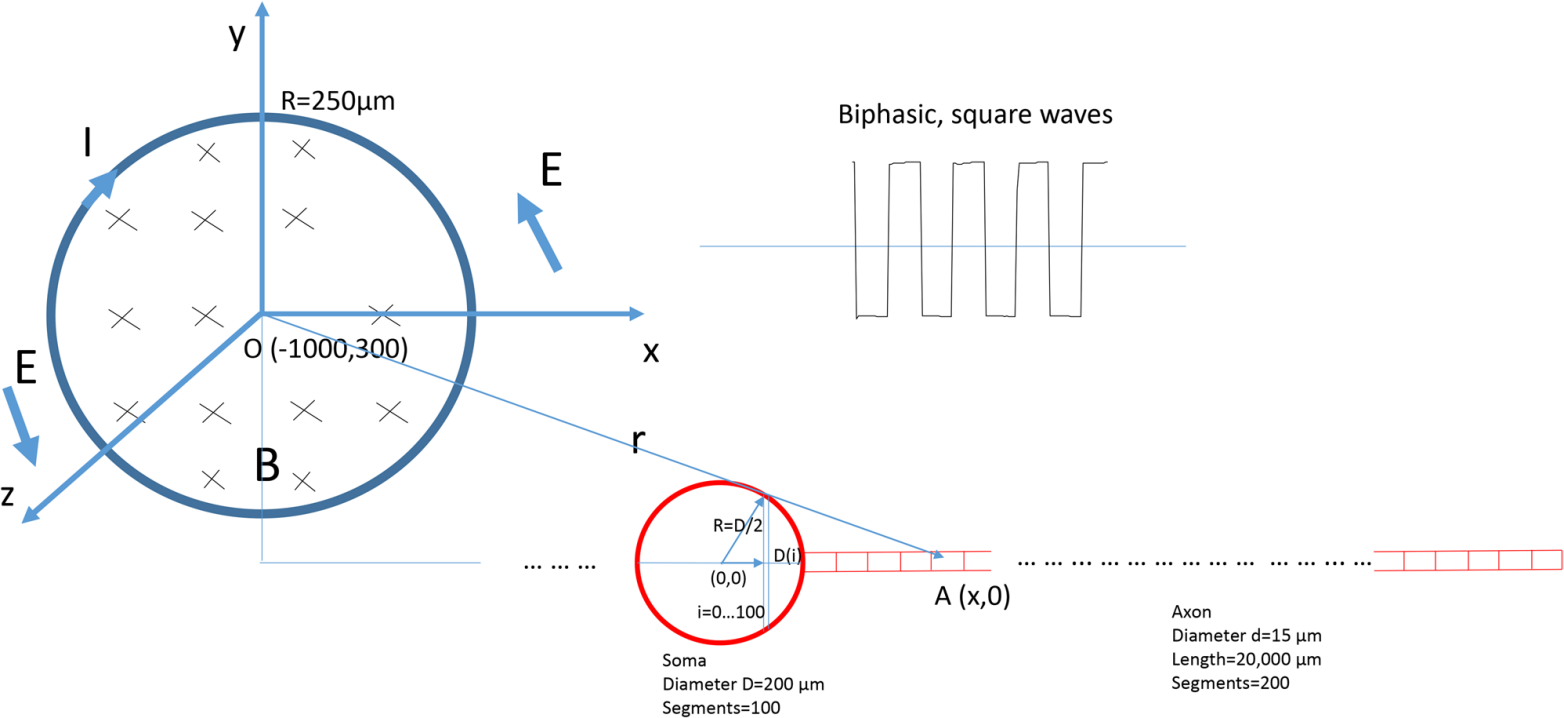
NEURON model for an *Aplysia* buccal neuron under miniature coil stimulation. The modeled neuron contained a spherical soma and a cylindrical axon. The soma sphere was 200 μm in diameter and was divided into 100 segments. The axon cylinder was 15 μm in diameter and 20,000 μm in length and was divided into 200 segments of equal length. Each neural compartment was modeled by H/H type ion channel mechanisms. The center of the soma was set to be (0, 0). The neuron was stimulated by a circular coil, whose center was located at (− 1000 µm, 300 µm). The radius of the coil was 250 μm. High frequency, biphasic electric pulses were delivered into the coil to induce electric field (E). I: coil current. X: direction of the magnetic flux when the coil current was increasing in a clockwise direction. Point A (x, 0) was an arbitrary point on the neuron, whose distance to the center of the coil is r in Eq. (2).

Effects of the high-frequency stimulation by the miniature coil were tested with a multi-compartment neuron model (Fig. 9) using NEURON simulation environment package^39^. The modeled neuron contained a spherical soma and an axon as a cylinder (Table 1). The Hodgkin/Huxley (H/H) type of dynamics of the fast sodium, slow potassium, and leakage channels in the membrane were inserted into the nodes^40^. Detailed electrical parameters (Table 2) of the modeled neuron were adapted from a published model of *Aplysia* buccal neuron^21^.

**Table 1:** Geometrical parameters of the NEURON model for an *Aplysia* neuron that contains a soma and an axon.

Geometric parameter	Value
Soma diameter	200 µm
Number of soma segments	100
Axon diameter	15 µm
Number of axon segments	200
Length of axon segments	100 µm
Total axon length	200,00 µm
Location of coil center (x_coil_)	− 1000 µm
Distance of coil center to axon (y_coil_)	300 µm

**Table 2:** Electric parameters of the NEURON model for an *Aplysia* neuron that contains a soma and an axon.

Electrical parameter	Value
Membrane capacitance (*C*_m_)	1 μF/cm^2^
**Fast Na + channels**	
Max. sodium conductance (g_Na__) in the soma	0.024 S/cm^2^
Max. sodium conductance (g_Na__) in the axon	0.12 S/cm^2^
Activation term (α_m_) of m gates	− (0.1ν + 4)(exp(− 0.1ν − 4)) − 1)^−1^
Inactivation term (β_m_) of m gates	4exp (− (ν + 65)/18)
Time constant of (t_m_) m gates	3(α_m_ + β_m_)*3^(t/10 − 2.0)^−1^
Activation term (α_h_) of h gates	0.07exp(− 0.05ν − 3.25)
Inactivation term (β_h_) of h gates	1/(exp(− 0.1ν − 3.5) + 1)
Time constant of (t_h_) h gates	1.7 ((α_h_ + β_h_)*3^(t/10 − 2.0))^−1^
Reversal potential (*E*_Na_)	50 mV
**Slow K + channels**	
Max. conductance (g_K__) in the soma	0.0072 S/cm^2^
Max. conductance (g_K__) in the axon	0.036 S/cm^2^
Activation term (α_n_) of n gates	− (0.01ν + 0.55) (exp(− 0.1ν − 5.5) − 1)^−1^
Inactivation term (β_n_) of n gates	0.125exp(− (ν + 85)/80)
Time constant of (t_n_) n gates	5.6((α_n_ + β_n_)*3^(t/10 − 2.0))^−1^
Reversal potential (*E*_K_)	− 77 mV
**Leakage channels**	
Conductance (*g*_L_)	0.00028 S/cm^2^
Reversal potential (*E*_L_)	− 65 mV

t: environmental temperature in Celsius.

v: membrane potential of a neural segment.

**Figure 10:**
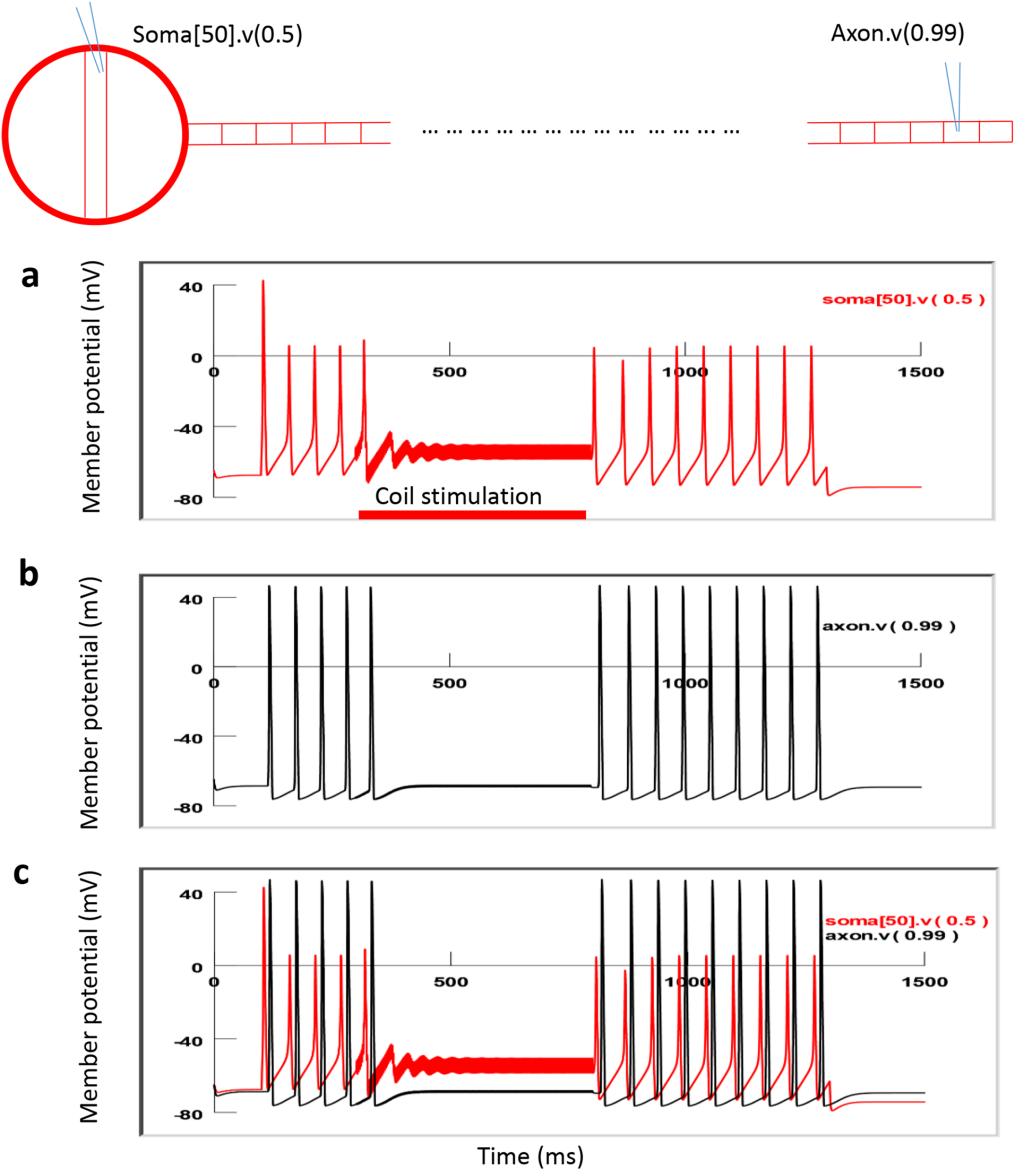
HFS with miniature magnetic coil inhibited soma activity and blocked neuron output in the modeled axon. The miniature coil was positioned at (− 1000, 300). A depolarization current was injected into the soma to trigger action potentials. A 500 ms train of biphasic square pulses (400 Hz) was applied to the coil, leading to the blockage of the action potentials in the soma. Activity recorded from the distal end of the axon was also eliminated. (**a**) Membrane potential recorded from the soma. (**b**) Membrane potential recorded from the distal axon. (**c**) Dual recording from the soma and the axon.

During stimulation, the action potentials were initiated in the soma by a constant depolarization current delivered by an intracellular electrode. These action potentials propagated along the axon to its distal end. For magnetic stimulation, the coil was located close to the soma with the center at (− 1000, 300) (Fig. 9). We applied 500 ms, 400 Hz magnetic stimulation to the axon. The shape of the square pulses was bi-phasic, similar to the measured extracellular potentials around the coil. To stimulate the neuron, the magnitude of the extracellular voltage (Eq. 3) was applied to the NEURON model^41^. The model was run at room temperature. Under strong, supra threshold stimulation, soma activity was rapidly and reversibly suppressed (Fig. 10a). The output of the neuron, as recorded from the distal end of the axon, was also eliminated (Fig. 10b). Therefore, NEURON modeling confirmed the soma inhibition and blocking of the neuronal output by the miniature coil which delivered high-frequency stimuli.

### Cellular and ionic mechanisms underlying magnetic inhibition of soma activity

What made the neuron fail to fire action potentials during magnetic stimulation? Excessive membrane potential changes can impair the capability of neurons to fire action potentials. For example, excessive depolarization can cause inactivation of the voltage-dependent sodium channels^42^. It is well known that extracellular current flow into the soma will hyperpolarize the soma, while current flow out of the soma will depolarize it, regardless if the electric current is applied by an electrode^21^, or by magnetic induction^43^. Under high-frequency stimulation, the bi-phasic stimulation generated an altered electric field that penetrated the soma membrane. Since the electric current penetrating the cell membrane alternated quickly, membrane polarization became insignificant. Therefore, suppression of the soma firing under high-frequency magnetic stimulation cannot be simply explained by membrane potential change. As a matter of fact, we did not observe significant membrane potential changes in the soma under magnetic stimulation (Fig. 10).

**Figure 11:**
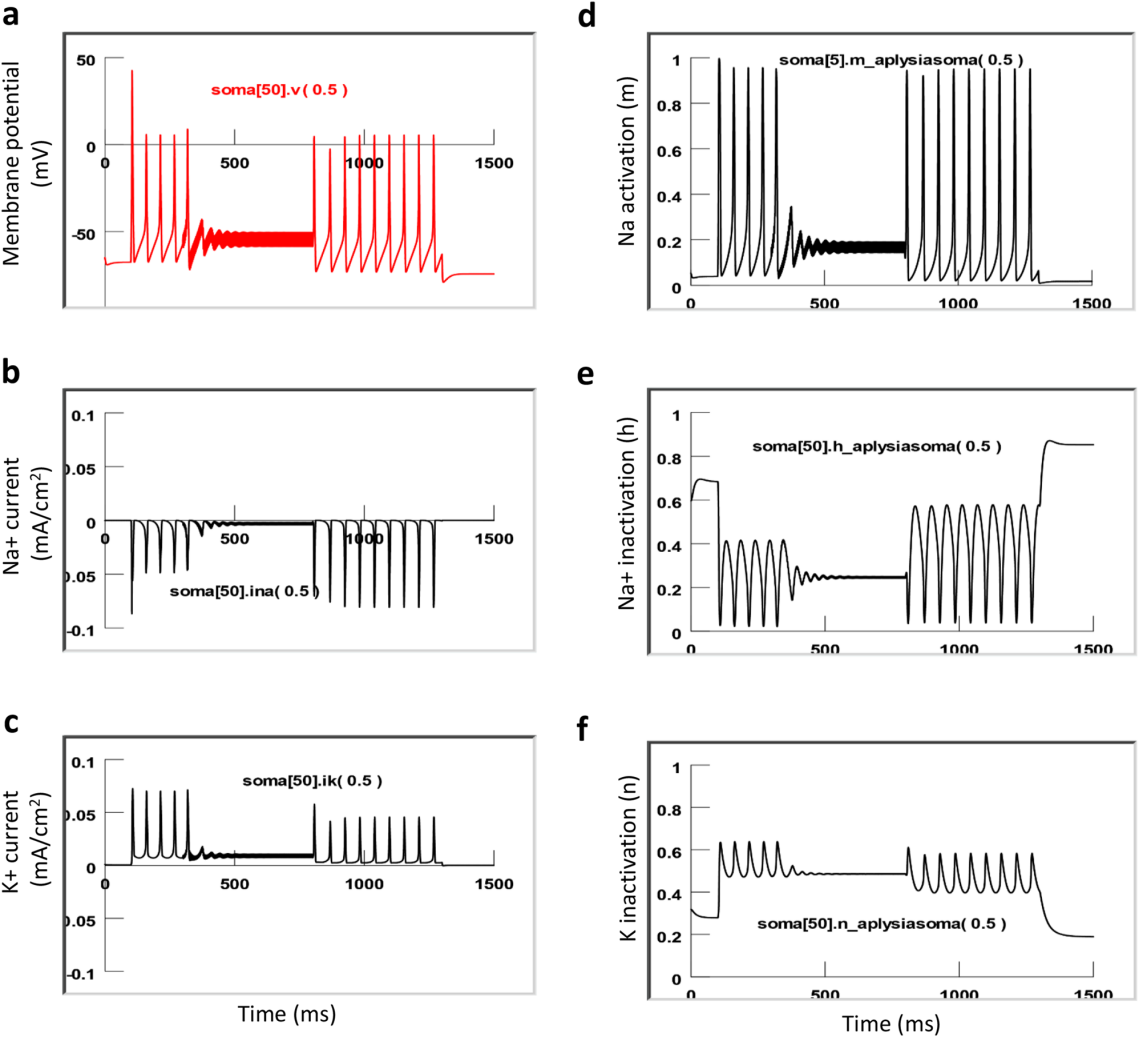
Ion channel dynamics underlying soma inhibition by high-frequency magnetic stimulation with the miniature coil. Membrane potential (**a**), Na^+^ current (**b**), K^+^ current (**c**), sodium channel activation m (**d**), sodium channel inactivation h (**e**) and potassium channel activation n (**f**) in the center of the soma (soma [50]) were plotted.

To further investigate the ionic mechanisms underlying high-frequency inhibition, we plotted the inward sodium current (INa^+^), outward potassium current (IK^+^), sodium channel activation (m) and inactivation (h) variables, and potassium channel activation variable (n, Fig. 11). In the absence of coil stimulation, the membrane was at resting potential (− 65 mV). Slight depolarization of the soma elicited constant firing of the soma. The sodium channel was modestly de-inactivated (h = 0.4) before the firing of each action potential. This allowed for full activation of the sodium channels (m = 1) to produce a large inward sodium current (Ina) and depolarization of the membrane for spiking. Meanwhile, activation of the potassium was substantial (n = 0.65), and a large inward potassium current was observed to hyperpolarize the membrane during the falling phase of the action potentials.

Under supra-threshold magnetic stimulation, membrane potential was slightly depolarized. Coil stimulation caused partial activation (m = 0.2) of the sodium channels and insufficient de-inactivation of the sodium channels (h = 0.25). These modulated ion channel dynamics led to a diminished inward current (Ina) and a failure of firing action potentials during the HFS coil stimulation. The coil stimulation also prevented the full activation of the potassium channel (n = 0.5) and diminished the outward current (Ik). Post-stimulus, the soma resumed firing capability under depolarization currents. We observed some interesting post-stimulus effects, including a slight hyperpolarization in the cell membrane and an increased sodium current. The sodium channels were sufficiently de-inactivated before the firing of another action potential (h = 0.6). In conclusion, coil stimulation at a high frequency caused insufficient sodium channel activation and de-inactivation, leading to the failure of sustaining the somatic action potentials.

## Discussion

Determining the site of stimulation is essential in estimating the outcome of neural stimulation with magnetic coil^44^. Recent developments of the miniature coil technology allow us to explore the possibility of ganglion cell control with magnetic stimulation. Simultaneous intracellularly recording from the soma and extracellular recording from the axon (far away from the coil) confirmed reliable inhibition of the soma by the miniature coil stimulation. NEURON model provided mechanistic insights on the ion channel dynamics underlying the magnetic inhibition of soma activity. There are four major finds coming from the study.

First, this paper is the first to demonstrate that by delivering high-frequency stimulus directly to the ganglion cell, the miniature sized coil could completely inhibit soma activity in *Aplysia* neurons (Figs. 3, 4, 5). Magnetic inhibition was independent of the firing state of the neurons (Figs. 3, 4). Magnetic inhibition was also effective in hyper-active neurons under the high potassium treatment (Fig. 5). This inhibitory effect on the ganglion cells was generic since many neurons (i.e., B3, B4/ B5, and B6) have been inhibited by the miniature coil.

Second, this paper is the first to demonstrate that somatic inhibition by the coil prevented the functional output from the targeted neuron (Fig. 6). By definition, B3 is a motor neuron that innervates the I1/I3 jaw muscle^28,29^. Recording from the distal end of the B3 neuron confirmed that motor output from this neuron was inhibited by the coil. Miniature coil stimulation on the ganglion was therefore local since it did not provoke unwanted neural activity in the axon.

Third, this paper is the first to demonstrate that presence of the ganglion sheath provides minimal alteration to the magnetically-induced electrical field. The coil could apply its inhibitive effects through the sheath of the buccal ganglion (Fig. 7). This may suggest a substantial benefit for clinical implantation with minimized invasion to the targeted ganglia.

Fourth, this paper provides a quantitative biophysics model that confirms that the miniature coil indeed induced a sufficiently large electric field on the single neuron (Fig. 8). Magnetic stimuli can therefore be optimized based on the geometric and electric parameters of the commercial coil, and the input signal applied to the coil. The NEURON model linked these parameters with the behavior of the soma activity (Fig. 9). NEURON modeling also provided insight into the ion channel dynamics for magnetic inhibition of ganglion cell activity (Fig. 11), mainly the sodium channel activation and inactivation processes. Further coil design could therefore be based on these analyses, to develop more powerful and efficient micro-coil technology for ganglion cell control.

### Implication for the further development of the micro-coil technology for ganglion cell inhibition

#### High-frequency stimuli for soma inhibition

Previous work suggested that anodic current that flows into the cell membrane provides hyperpolarization effects and inhibits neuron activity^33^. When applied for soma inhibition, cathodic currents were used to inhibit the neuron when the electrode was positioned close to the soma opposite to the axon^21^. However, a coil can't produce a constant electric field for a long period of time, since this will require a continuous increase of electric current inside the coil (Faraday’s law).

Previous work demonstrated that high frequency, AC electric currents were effective in soma^45^ and axon^46^ inhibition. However, delivering high-frequency pulses using a large coil is challenging^47^, due to the technical difficulties encountered (much higher impedance, higher energy-storage requirement, and severe cooling issues). As a consequence, the effects of high-frequency stimulation on soma by magnetically-delivered electric current has rarely been explored.

The small sized coil is suitable for the delivery of high-frequency stimuli without generating obvious thermal effects, providing an interesting alternative to the conventional DC or high-frequency methods with electrodes for soma blockage. We have chosen this frequency (400 Hz) based on our preliminary observation that several hundred Hz of pulses were necessary for complete axonal inhibition^19^. If the frequency of the stimulus is too low (i.e., several Hz), it cannot cause complete blockage of the soma. We have simulated the effects of the low frequency stimuli to the soma, and found that low frequency stimuli couldn’t cause sustained changes in the ion dynamics, which was crucial for the blockage of soma activity at 400 Hz (Fig. 11).

#### Spatial specificity in miniature coil stimulation

One major challenge for coil-based stimulation is its poor spatial specificity. Large coils could not provide selective stimulation and may affect many unwanted neurons and lead to unpredictable results such as inducing seizures in the brain^48^. The miniature coil could be implanted to the targeted area to improve focal stimulation.

In our study, since the size of the buccal ganglion was comparable to the size of the coil, it was difficult for the current coil to achieve single cell blockage but spare others. However, we did observe certain level of specificity achieved by the coil. First, when the coil was positioned next to the buccal ganglion, its inhibitory effects were constrained to the cell body, but not the axon. Second, effective inhibition was most likely happening in neurons next to the coil. Overall, we have found that high frequency stimulation was inhibitory to all the tested neurons in a specific area close to the coil, including large motor neurons B3, B6 and interneuron B4/B5 neurons in the buccal ganglion (Fig. 2). There neurons locate on one side of the caudle surface and play a significant role in contracting the jaw muscles in *Aplysia californica*^23^. The other side of the ganglion contains a cluster of very small sensory neurons (Fig. 2, also see^35^) and was relatively far away from the coil (around 1 mm from the coil center). Since the coil-induced electric field decayed quickly with distance (Fig. 8), electric field intensity applied to these sensory neurons was about 30% of those to the B3 neuron. It is possible, therefore, that these neurons are less sensitive to the magnetic stimulation. Since it is extremely difficult to intracellularly record from these tiny sensory neurons, we have ran a simulation to test this hypothesis. The small neuron was assumed to have a small size (10 µm in diameter), and was positioned much farther away from the coil by assigning the coil center at x = -1000 µm and y = 1000 µm. We found that high frequency simulation could not blockage the small neuron in far distance (data not shown), although it could suppressed large neuron B3 in short distance (Figs. 3, 4, 5, 6, 7).

To increase the specificity, a novel design of the miniature coil shall aim at further decreasing the size of the coil, and increasing the local gradients of the electric field (defined as “activating function”^49^). Decreasing the size of the coil allows for a closer positioning of the coil next to the targeted neuron. The gradients of the electric field determines the location and speed of depolarization or hyperpolarization by the extracellular stimulation^50^. Taking advantage of this understanding, strategies for increasing the coil curvature and field gradient have been proposed. For example, bending the coil wire to form a sharp angle has been used for single neuron activation^15^, since this could create a local site, where the gradient of the electric field was maximal.

#### Trans-sheath stimulation by the miniature coil

When an electrode was used for neural stimulation, the electric current flows through the inhomogeneous tissue surrounding the electrode and sometimes failed to reach the targeted neuron. For example, this shunting effect was observed in the spinal cord stimulation, where electric currents flow to the conductive cerebrospinal fluid^51^. Here we show that membrane ganglion sheath did not affect the coil’s efficacy in soma inhibition, due to the fact that the electric current flow inside the ganglion is induced by the coil outside the ganglion, which avoided the above-mentioned shunting effect. Since the biological tissue, such as the ganglion sheath, has very high permittivity at a frequency of several hundred hertz^52^, magnetic field can penetrate into the buccal ganglion without too much attenuation. This ensures large enough electric field to be generated inside the ganglion for neural inhibition. Theoretically, ganglion cell stimulation with a miniature coil shall provide a more controllable and consistent outcome. The ganglion sheath can provide an attachment point for the positioning of the miniature coil in future in vivo experiments.

#### Biocompatibility in miniature coil stimulation

One technique problem associated with electric stimulation with a chronically-implanted electrode is the conductivity changes in the medium surrounding the electrode, which could affect the neural response^49^. This conductivity change could be due to pathological conditions such as inflammation reaction of the tissue^16^, and glial scar formation^53,54^. It could also be due to the oxidization of the electrode, making electric stimulation less consistent. During magnetic field stimulation, the magnetically-induced electric field is mainly dependent on the biophysical properties of the inhomogeneous tissue surrounding the coil. Therefore, a major advantage of the coil method is that stimulation performance could be sustained even though the coil is encapsulated by gliosis due to foreign body reactions. Furthermore, coating the coil with biocompatible, soft, dielectric material will not alter the induction of the local electric field and outcome of stimulation. In conclusion, the miniature coil can provide improved biocompatibility and consistency than the traditional metal electrodes for chronic implantation and neural stimulation.

#### Potential thermal effects

Optimal selection of stimuli (frequency and magnitude) for magnetic inhibition of ganglion cells shall consider the potential thermal effects generated by the coil, which could damage the coil or introduce irreversible tissue damage. In our experiments, the electric current that flowed inside the miniature coil was approximately 1.08A, a value much lower than what could damage the coil (~ 10 A^18^). This current generated a less than 3 °C of temperature rise in a local area next to the coil. In our experiments, neural activity could always recover after magnetic inhibition (Figs. 3, 4, 5, 6, 7), suggesting no neural damage was caused by the coil stimulation. For mammalian myelinated nerves in cats, heat blockage on action potentials happens at around 50 °C in^55^. Invertebrate axons operate between 0 and 40 °C^56^, and a more than 20 °C of local temperature increase was needed for thermal blockage of the unmyelinated squid giant axon^57^. Therefore, we excluded the possibility of thermal blockage of ganglion cells and potential damage in our experiments.

### Limitations of the NEUORN model and future work

There are several limitations in the modeling work that could be improved in the future. First, the geometry of the coil is oversimplified as an infinitely long cylinder in the biophysical estimation of the induced electric field by the coil. Finite element modeling could provide a more accurate representation of the coil shape and electric field calculation^41^. Second, the Hodgkin/Huxley-based soma model did not include several ionic channel mechanisms that might be affected by the magnetic stimulation, such as Na^+^ channels, Ca^2+^ channels and A-type K^+^ channels that are essential for neural excitability^58^. High-frequency stimulation could cause depolarization blockage, via excessive K^+^ ionic accumulation in the extracellular space^59,60^. This possibility can be confirmed by direct recording of the K^+^ level and incorporated into the model. Finally, the single neuron model does not include synaptic impacts from other neurons, which could also be affected by the magnetic fields^61^.

### Clinical implications of the coil inhibition on ganglion cells

This work illustrates the cellular responses when ganglion neurons were inhibited by a miniature coil. Regardless of the exact mechanisms, results from this paper suggest several potential areas of clinical applications for this technology.

#### Dorsal root ganglion stimulation

Primary sensory neurons generate spontaneous activity in a healthy state. Following nerve injury, there is a substantial increase in ectopic activity generated by nociceptive neurons within the dorsal root ganglion^62,63^. This sensitization and hyper-excitability of the sensory neurons in the dorsal root ganglion leads to neuropathic pain^8^. Electric stimulation that targets on inhibition of the primary sensory neurons that anatomically innervate the painful areas could provide a faster and specificity for pain blockage^64–66^. Complications in such DRG stimulation involve lead erosion and damage and lead connection failure. The procedural would also cause dura puncture and infection^67^. A miniature coil could be potentially used for DRG stimulation to suppress ectopic activity and thereby provide analgesia. The coil may provide consistent application of electric current, and the soft, bio-compatible material cover the coil could minimize the potential injury to the DRG.

#### Retinal ganglion neuron stimulation

Retinal prosthetic devices restore sight in visually impaired people by means of electrical stimulation of surviving retinal ganglion cells (RGCs)^68,69^. By selectively stimulating RGCs near their somas and dendrites, one may achieve more localized phosphenes. As such, a miniature coil has been used to activate the retinal ganglion neurons by delivering strong, low-frequency pulses to the RGCs^18^. On the other hand, RGC hyperpolarization and inhibition is likely to be a significant contributor in forming retinal activation pattern^69^, which can be done with coil-based high-frequency stimulation, as suggested by this paper. It will be interesting to use the same miniature coil to achieve dual excitation/inhibition effects in RGCs.

#### Seizure control

Here we demonstrated single neuron inhibition by the miniature coil in a high K^+^ induced, hyper-excitable neuron (Fig. 5). It appears that the miniature coil provided local inhibition effects to both the soma (Fig. 5) and the axons^19^, and eliminated synaptic events (Fig. 5). The high potassium method has been widely used for studying pathological conditions such as seizures^32^. Recently, we found that high-frequency stimulation with the miniature coil could suppress epileptic form activity that was induced by high-K + solution in the hippocampus^20^, while the mechanisms are still unclear. The miniature coil technique could be further developed for seizure inhibition in the epileptic focal.

## Methods

### Miniature coil and the magnetic stimulator

Commercial multilayer surface mount inductors (100nH, MLG1005SR10JTD25, TDK U.S.A. Corporation, Uniondale, NY) were used for the study. The inductor has an inductance of 100 nH and resistance of 2 Ω. The internal structure of the coil (Fig. 1a) was visualized by removing the ceramic core and epoxy coating using 40% liquid hydrofluoric acid and 10 N HCL, based on a published protocol^20^. It was a 1 mm × 0.5 mm rectangle. The coil contained 20 loops and was 0.5 mm long. Two copper wires (magnetic wire 32-AWG, GC electronics, IL, L3-616) were soldered to the two metal leads of the inductor for electric current delivery (Fig. 1b). To ensure electrical insulation and water impermeability of the exposed coil terminals during electrophysiological experiments, the coil was then coated with acrylate copolymer enamel (Revlon, New York)^70^. The magnetic stimulator^19,20^ was composed of an arbitrary function generator (AFG1022, Teletronix), which generated a stimulation signal. The signal triggered large current pulses through a 1000 W power amplifier (Pyramid PB 717X 2 channel, Pyramid Car Audio, Brooklyn, NY, 11204), which flew through the miniature coil. The amplifier was powered by a Triple Channel DC Power Supply (2231A-30-3, Keitheley).

### In vitro Aplysia electrophysiology

In total, 30 animals were used for the study. *Aplysia californica* (100–150 g) were obtained from Marinus Scientific (Newport Beach, CA) and were kept in the artificial seawater at room temperature (20 ± 1 °C). Animals were anesthetized by an injection of isotonic MgCl_2_ (50% of body weight). The buccal ganglion was dissected and immersed in an *Aplysia* saline solution (pH 7.4), which contained 460 mM NaCl, 55 mM MgCl_2_·H_2_O, 11 mM CaCl_2_·2H_2_O, 10 mM KCl, and 10 mM Hepes.

For soma stimulation and recording using extracellular electrodes, the buccal ganglion was pinned done with caudal side up, and was partially desheathed by peeling off the top layer of the sheath with fine dissection tools. This allowed the visualization of the individual soma (Fig. 2) and closer positioning of the extracellular electrode to the targeted neuron. The extracellular electrodes were made by pulling single-barreled capillary glasses using a Flaming-Brown micropipette puller (P-30, Sutter Instrument). For extracellular recording from the soma, the size of the electrode tip was adjusted to be slightly smaller than the size of the cell bodies^21,23^. Electrodes made in the same pulling protocol were also used for nerve suction recording^71^ from buccal nerve II (BN2). Since the electrode tip was smaller than the diameter of the nerve, we broke this tip so that the nerve end could fit into the glass capillary. Extracellular recordings were amplified by a Model 1700 differential AC Amplifier (A-M Systems) with a gain of 1000 and filtered by a 1.0–500 Hz band pass filter.

For soma stimulation and recording with intracellular electrodes, the buccal ganglion was completely de-sheathed to expose the cell bodies. All experiments were performed at room temperature. The intracellular electrodes were also made by pulling single-barreled capillary glasses using a Flaming-Brown micropipette puller. The pulling protocol was adjusted so that the tip of the electrode was sharp for cell penetration. Sharp electrodes were backed filled with 3 M potassium acetate before use. Intracellular signals were amplified using a DC-coupled amplifier (model 1600, A-M systems). DC offset was eliminated and the bridge was balanced for stimulation and recording. To control the frequency of firing of recorded neurons, an isolated pulse stimulator (model 2100, A-M systems) was connected to the 1600 amplifier to deliver short pulses. Both intracellular and extracellular signals were digitized (25 kHz) by a CED 1401, recorded, and analyzed by Spike 2 software (version 7.2, Cambridge Electronic Design Limited).

The concentration of the extracellular K^+^ in normal *Aplysia* saline was 10 mM. In some experiments, high concentration K^+^-containing *Aplysia* saline (24 mM) was added to the petri dish to excite the buccal ganglion. The final concentration of the K^+^ was calculated based on

1 $$ \left[ {K + } \right]_{0}  = \left( {V_{1} \left[ {K + } \right]_{1}  + V_{2} \left[ {K + } \right]_{2} } \right)/\left( {V_{1}  + V_{2} } \right) $$

where V_1_ and [K +]_1_ were the original volume and K^+^ concentration (10 mM) of the *Aplysia* solution in the dish, respectively. V_2_ and [K +]_2_ were the volume and K + concentration of the solution added into the dish, respectively. In the experiments, V_2_ = 50% V_1_, and the final concentration [K +]_0_ was 14.7 mM.

### Miniature coil stimulation on ganglion neurons

To position the miniature coil close to the targeted ganglion cell for stimulation, we inserted the two copper wires that were attached to the coil through a glass pipette (TW150F-4, WPS). We mounted the glass pipette on a micromanipulator for the positioning of the coil. We have positioned the coil close to ganglion side (caudal side up) where large motor neurons locate (Fig. 2). The coil was orientated so that its induced electric field was in parallel to the ganglion – BN2 axial (Fig. 3a), to generate effective stimulation^36,38^. 400 Hz, bi-phasic square waves were delivered to the power amplifier for the stimulation for about 10 s. We found that when square waves with a frequency > 50 Hz were delivered to the coil, the output voltage across the coil maintains was also square waves. For a 10 V input signal, it generated a 2.16 V output crossed the coil. The waveform of the coil-induced electric potential was measured in the dish by positioning an extracellular glass electrode next to the coil.

The impedance of the coil was measured at the beginning and end of each experiment to test its connectivity. Potential leakage of the coating coverage was also tested by measuring the impedance of the coil to the ground. If present, this current generated an extremely large level of noise. The local temperature around the coil was measured with a thermocouple, which was connected to a digital thermometer (HH11B, Omega Engineering, Norwalk, CT) to display the temperature with 0.1 °C resolution.

### Computation of electric potential around the miniature coil

The induced electric field generated by an infinitely long, circular shaped coil was calculated by (Skach et al.^19^)

2 $$ E = \frac{{R^{2} }}{{2r}}\frac{{v\mu _{0} N}}{{Ll}}\quad \left( {r > R} \right) $$

where r represented the distance between the center of the coil to a point outside the coil. R was the radius of the coil, v was the instantaneous voltage across the inductor, L was the inductance of the coil in Henry, N was the loop number and l was the length of the coil, respectively. *μ*_0_ = 4π × 10^−7^ H/m was the vacuum permeability.

Electric potential distribution along the soma-axon axial was expressed as (Skach et al.^19^)

3 $$ V\left( x \right) = \frac{{V\mu _{0} NR^{2} }}{{2Ll}}{\text{atan}}\left( {\frac{{x - x_{{coil}} }}{{y_{{coil}} }}} \right) $$

where (x, 0) was defined as the location of an arbitrary point (A) on the modeled cell, and (x_coil_, y_coil_) was the center of the coil (Fig. 9a). This extracellular voltage distribution was applied to an *Aplysia* neuron model using NEURON simulation environment for soma blockage.

### Multi-compartment NEURON model of an *Aplysia* neuron

Effects of the high-frequency stimulation with miniature coil on the soma were tested with a multi-compartment soma-axon model using NEURON simulation environment package^39^. The model contained a spherical soma and a cylindrical axon (Table 1). The diameter of the soma (D) was 200 µm, matching the size of the B3 neuron. The soma was divided into 100 (N, i = 0–99) segments along its soma-axon axis. Each segment was 2 µm in length and was in the shape of a cylinder-disk. The soma tip segment (i = 0) was set to be 1 µm in diameter. The diameters of the rest of the soma disks, D(i) (i = 1–99), were computed as a function of its distance to the center of the soma (Fig. 9).

4 $$ D\left( i \right) = \sqrt {\left( {\frac{D}{2}} \right)^{2}  - \left[ {\left( {\frac{N}{2} - i} \right)\frac{D}{N}} \right]^{2} } $$

The neuron contains a single axon with a diameter of 15 µm and a length of 20,000 µm. The axon was divided into 200 segments evenly and each segment was 100 µm in length (Fig. 9).

The Hodgkin/Huxley (H/H) type of the fast sodium, slow potassium, and leakage channels in the membrane were inserted into the nodes^40^. The ionic current (I) at the n-th segment of the axon was described as

5 $$ I_{n}  = g_{{Na}} m^{3} h\left( {V_{n}  - V_{{Na}} } \right) + g_{k} n^{4} \left( {V_{n}  - V_{k} } \right) + g_{L} \left( {V_{n}  - V_{L} } \right) $$

where V_Na,_ V_K,_ and V_L_ were the equilibrium membrane potentials for sodium, potassium, and leakage channels, respectively. gNa, gk and gL were maximal conductance of Na, K, and leakage channels, respectively. m, h and n were dimensionless variables, whose values change between 0 and 1. m and h represented the activation and inactivation of the sodium channels, whereas n represented activation of potassium channels, respectively. The evolution equations for variables m, h, and n were:

6 $$ \frac{{dm}}{{dt}} = \alpha _{m} \left( {1 - m} \right) - \beta _{m} m $$

7 $$ \frac{{dh}}{{dt}} = \alpha _{h} \left( {1 - h} \right) - \beta _{h} h $$

8 $$ \frac{{dn}}{{dt}} = \alpha _{n} \left( {1 - n} \right) - \beta _{n} n $$

Detailed electrical parameters of the modeled soma and axon (Table 2) were adapted from a published model of *Aplysia* buccal neuron^21^. To simulate the lower densities of Na + and K + channels in the soma compared to the axon, the maximal conductance of Na + and K + channels in the soma were set to be 1/5 of those in the axon^21^. The time constants of the Na + and K + channels were increased by linear scaling factors based on the ratios of the time constants of the Hodgkin-Huxley model to the time constants of *Aplysia* sensory neurons^72^.

To simulate the effect of axonal blockage by the miniature coil, action potentials were initiated in the soma of the modeled neuron by injecting a small constant depolarizing current. A recording electrode was positioned in the center segment of the soma (soma [50]) to record soma activity. Another electrode was positioned in the distal end of the axon (Axon [0.99]), to record the output of the neuron from its distal axon.

The simulation was run for a total of 1500 ms. Simulation was started by setting the membrane potential at -65 mV. At 100 ms, a depolarization current (1200 ms in duration) was injected into the soma to elicit action potentials. At 300 ms, the 400 Hz, bi-phasic square waves were programmed and applied to the coil for a total of 500 ms. The coil was positioned at (-1000 µm, 300 µm). This represented the close proximity of the coil to the soma, while also taking into account the cover material on the coil (Fig. 9). To simulate the coil stimulation, the electric voltage induced by the miniature coil (Eq. 3) was applied to the modeled NEURON^41^. The model simulation was performed at room temperature (20 °C), as in the electrophysiological experiments.

## Data Availability

All data generated or analyzed during this study are included in this published article.
